# Consensus of Expert Opinion for the Diagnosis and Management of Hypermanganesaemia With Dystonia 1 and 2

**DOI:** 10.1002/jimd.70031

**Published:** 2025-05-04

**Authors:** Sherry Fang, Peter T. Clayton, Divyani Garg, Sangeetha Yoganathan, Maha S. Zaki, Elin A. Helgadottir, Vala K. Palmadottir, Maude Landry, Sidney M. Gospe, Kshitij Mankad, Vincenzo Bonifati, Suvasini Sharma, Karin Tuschl

**Affiliations:** ^1^ Department of Metabolic Medicine Great Ormond Street Hospital for Children London UK; ^2^ Department of Genetics and Genomic Medicine UCL Great Ormond Street Institute of Child Health, University College London London UK; ^3^ Department of Neurology All India Institute of Medical Sciences New Delhi India; ^4^ Paediatric Neurology Unit, Department of Neurological Sciences Christian Medical College Vellore India; ^5^ Department of Clinical Genetics Human Genetics and Genome Research Institute, National Research Centre Cairo Egypt; ^6^ Landspitali University Hospital Reykjavík Iceland; ^7^ The Moncton Hospital, Horizon Health Network Moncton Canada; ^8^ Department of Neurology and Pediatrics University of Washington Seattle Washington USA; ^9^ Department of Pediatrics Duke University Durham North Carolina USA; ^10^ Department of Radiology Great Ormond Street Hospital for Children London UK; ^11^ Erasmus MC, University Medical Center Rotterdam Rotterdam the Netherlands; ^12^ Department of Pediatrics Lady Hardinge Medical College and Associated Kalawati Saran Children's Hospital Delhi India

**Keywords:** consensus recommendations, HMNDYT1, HMNDYT2, manganese, SLC30A10, SLC39A14

## Abstract

Hypermanganesaemia with Dystonia 1 and 2 (HMNDYT1 and 2) are inherited, autosomal recessive disorders caused by pathogenic variants in the genes encoding the manganese transporters SLC30A10 and SLC39A14, respectively. Impaired hepatic and enterocytic manganese uptake (SLC39A14) and excretion (SLC30A10) lead to deposition of manganese in the basal ganglia resulting in childhood‐onset dystonia‐parkinsonism. HMNDYT1 is characterized by additional features due to manganese accumulation in the liver causing cirrhosis, polycythaemia, and depleted iron stores. High blood manganese levels and pathognomonic MRI brain appearances of manganese deposition resulting in T1 hyperintensity of the basal ganglia are diagnostic clues. Treatment is limited to chelation therapy and iron supplementation that can prevent disease progression. Due to their rarity, the awareness of the inherited manganese transporter defects is limited. Here, we provide consensus expert recommendations for the diagnosis and treatment of patients with HMNDYT1 and 2 in order to facilitate early diagnosis and optimize clinical outcome. These recommendations were developed through an evidence and consensus‐based process led by a group of 13 international experts across the disciplines of metabolic medicine, neurology, hematology, genetics, and radiology, and address the clinical presentation, diagnostic investigations, principles of treatment, and monitoring of patients with HMNDYT1 and 2.

## Graphical abstract

The presented evidence and consensus based recommendations provide a framework for the diagnosis and management of patients with hypermanganesaemia with dystonia 1 and 2 caused by pathogenic variants in *SLC30A10* and *SLC39A14*.

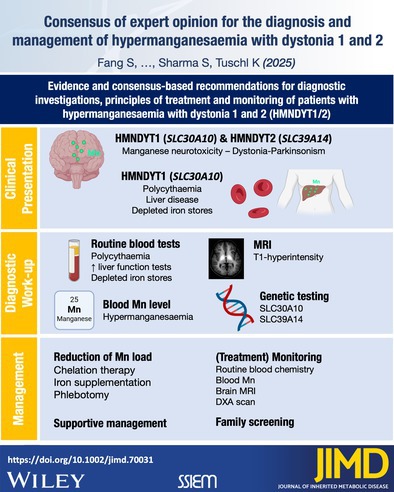

## 1 Introduction

**FIGURE 1:**
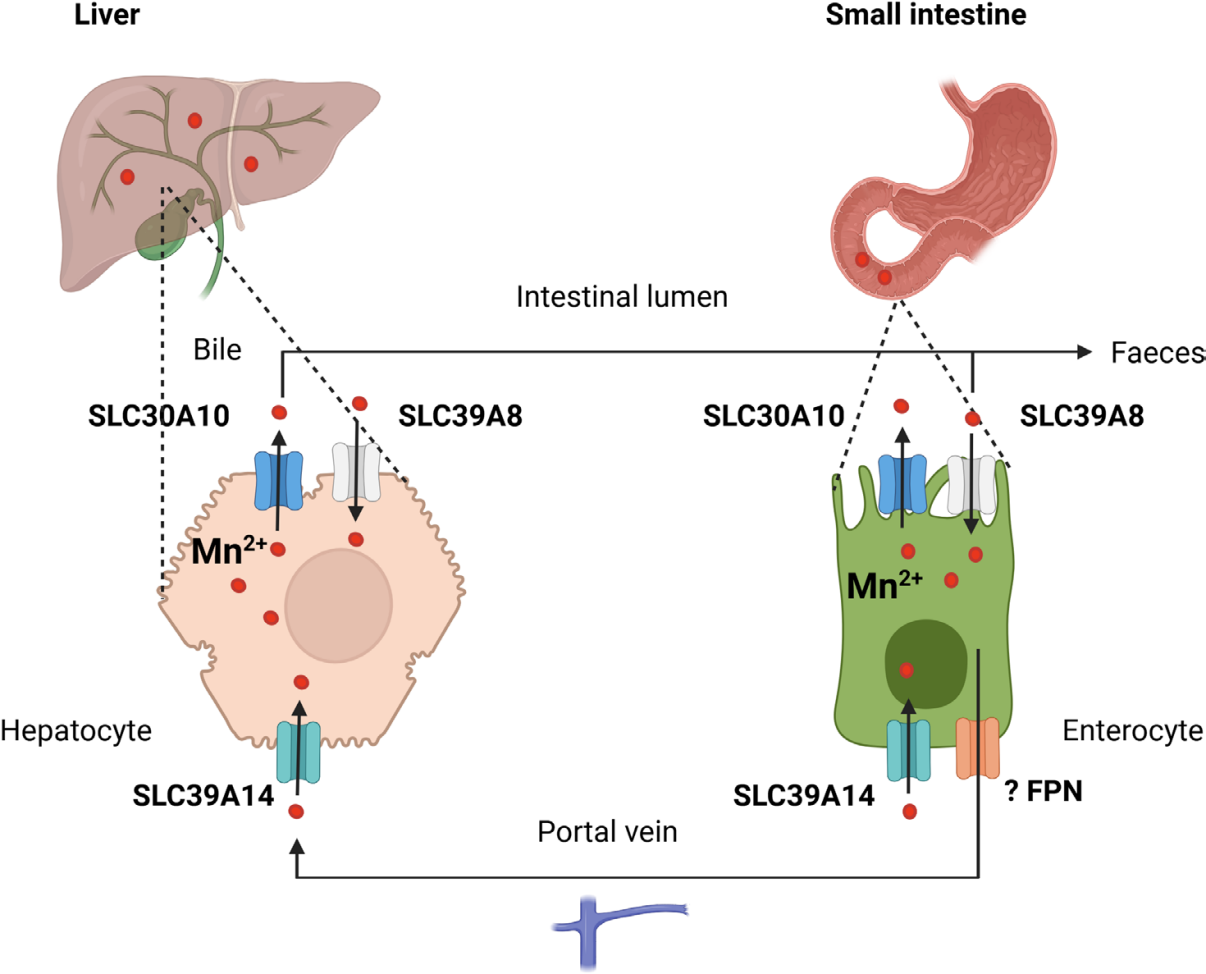
Solute carrier (SLC) transporters contributing to the maintenance of manganese homeostasis in humans. Schematic depicting the functions of SLC30A10, SLC39A14, and SLC39A8 that are expressed in both hepatocytes and enterocytes. SLC30A10, a manganese efflux transporter localized at the cell membrane, facilitates biliary and intestinal excretion of manganese. SLC39A14, a manganese uptake transporter expressed at the basolateral membrane of polar cells, shuttles manganese into hepatocytes and enterocytes for subsequent excretion via SLC30A10. SLC39A8 is a manganese uptake transporter expressed on the apical side of polar cells required for manganese uptake into the cell. Efflux into the blood at the basolateral membrane is likely to occur via ferroportin (FPN). Created with BioRender.com.

Manganese is an essential trace metal required for brain physiology and metabolism since it acts as a co‐factor for multiple enzymes [1, 2, 3]. Tight regulation of its homeostasis is crucial since both manganese deficiency and overload are detrimental to human health [4, 5]. In recent years, a network of SoLute Carrier (SLC) transporters has been identified that facilitates the maintenance of manganese homeostasis (Figure 1) [6, 7, 8, 9, 10]. SLC30A10 is a manganese efflux transporter expressed in hepatocytes and enterocytes that aids the excretion of manganese into the bile and intestine [7, 11]. SLC39A8 and SLC39A14, on the other hand, are manganese uptake transporters that are expressed on opposite sides of polar cells such as hepatocytes and enterocytes [8, 10, 12, 13]. While SLC39A8 on the apical membrane allows uptake of manganese into the organism, SLC39A14 on the basolateral membrane shuttles manganese across the cell for subsequent excretion via SLC30A10 [13]. Pathogenic variants in both *SLC30A10* and *SLC39A14* affect manganese transport in hepatocytes and enterocytes, resulting in reduced biliary/intestinal excretion and subsequently systemic manganese overload and neurotoxicity [7, 8, 14]. Biallelic pathogenic variants in *SLC30A10* cause hypermanganesaemia with dystonia 1 (HMNDYT1, OMIM #613280) that is characterized by childhood‐onset dystonia‐parkinsonism, chronic liver disease progressing ultimately to cirrhosis, polycythaemia, and depleted iron stores [7, 14]. Accumulation of manganese in the liver leads to activation of the hypoxia‐inducible factor (HIF) pathway reflected by increased erythropoietin synthesis and polycythaemia, as well as depleted iron stores [15]. Biallelic pathogenic variants in *SLC39A14*, on the other hand, cause hypermanganesaemia with dystonia 2 (HMNDYT2, OMIM # 617013) presenting with an isolated neurological phenotype characterized by rapidly progressive, childhood‐onset dystonia‐parkinsonism [8]. In both HMNDYT1 and HMNDYT2, chronic manganese overload leads to deposition of manganese in the brain, particularly the globus pallidus, striatum, and dentate nucleus, where it is neurotoxic and causes a distinct movement disorder similar to what is observed in environmental manganese overexposure, a condition known as manganism [16, 17]. Acquired manganism has been observed in industrial settings (mining and welding industries) following inhalation of manganese‐laden dust, upon overexposure due to contaminated well water, parenteral nutrition, or ephedrone preparations, or in end‐stage liver disease when impaired liver function results in insufficient manganese excretion [18, 19, 20, 21, 22]. Pronounced generalized dystonia leads to a characteristic high‐stepping, “cock‐walk gait” independent of the cause of manganese overload [23]. A multitude of molecular mechanisms have been suggested to contribute to manganese neurotoxicity that include oxidative stress, mitochondrial dysfunction, calcium dyshomeostasis, and endoplasmic reticulum stress [24, 25, 26, 27, 28, 29]. Manganese appears to exert its effect on multiple neuronal subtypes that include dopaminergic, glutamatergic, GABAergic, and cholinergic neurons, as well as astrocytes [19, 30, 31, 32, 33, 34, 35, 36, 37]. While some similarity exists between the Parkinsonian symptoms of manganism and Parkinson's disease, manganism has a distinct entity with absence of dopaminergic neurodegeneration [38]. Studies in murine models suggest that manganese impairs dopaminergic neurotransmitter release through molecular mechanisms that remain unidentified [39, 40].

Atypical presentations of HMNDYT1 include that of isolated polycythaemia [41, 42], spastic paraparesis [7, 43] as well as adult‐onset parkinsonism mimicking Parkinson's disease [14]. Milder cases of HMNDYT2 with onset of movement disorder in early adulthood have also been described [44].

SLC39A8 facilitates uptake of manganese in the small intestine and reclaims manganese from the bile, thus increasing the body's manganese load [13]. Biallelic pathogenic variants in *SLC39A8* lead to the only known manganese deficiency syndrome (Congenital Disorder of Glycosylation 2 N, OMIM #616721) [10, 45, 46]. The developed consensus recommendations do not apply to this disorder.

**FIGURE 2:**
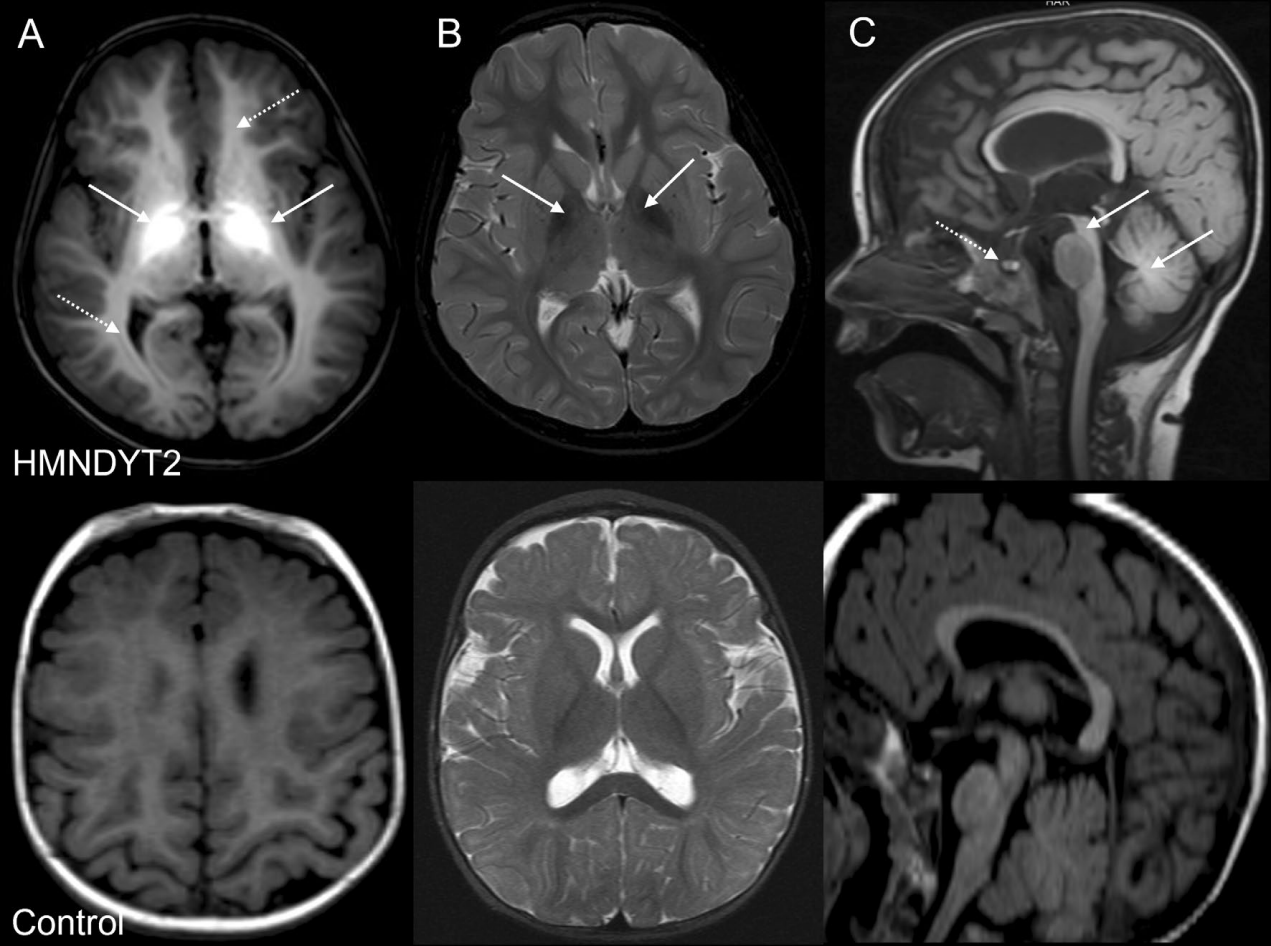
Characteristic MRI brain appearances of manganese deposition. Representative images from a 10 month old patient with HMNDYT2 (top row) compared to a healthy control subject (bottom row). (A) T1‐weighted imaging, axial view: T1‐weighted hyperintensity marked by a white arrow pointing to the globus pallidus, dashed arrow pointing to the white matter. (B) T2*‐weighted imaging, axial view: Corresponding hypointensity marked by a white arrow pointing to the globus pallidus. C T1‐weighted imaging, sagittal view: White arrows pointing to dorsal pons and cerebellar peduncles, dashed arrow pointing to T1 shortening in the anterior pituitary gland.

Diagnosis of HMNDYT1 and 2 is usually made through magnetic resonance imaging (MRI) of the brain due to the characteristic appearances of manganese deposition that include hyperintensity of the basal ganglia (consistent with the observed movement disorder), white matter, and pituitary gland on T1‐weighted imaging accompanied by hypointensity on T2‐weighted imaging (Figure 2) [8, 16, 47]. While T1 hyperintensity of the pituitary gland appears to be a consistent feature, endocrinopathies have rarely been described in HMNDYT1/2. Whole blood manganese levels are commonly hugely increased in both HMNDYT1 and 2 [48, 49]. Polycythaemia, impaired liver function tests, and iron deficiency further point to a diagnosis of HMNDYT1, and the absence of these features points to HMNDYT2 [7, 50].

*SLC30A10* is located on chromosome 1 encoding two protein coding transcripts, while *SLC39A14* is located on chromosome 8 encoding 11 protein coding transcripts. To date, more than 60 patients with biallelic pathogenic variants in *SLC30A10* and 33 in *SLC39A14* are stated in the literature, consistent with the autosomal recessive inheritance pattern of the two disorders. Further, five patients with characteristic clinical phenotype but undetermined genetical status have been reported (Table S1).

Differential diagnosis of the inherited manganese transporter defects HMNDYT1 and 2 includes acquired causes of manganese overload, also known as manganism. Manganese neurotoxicity can occur in occupational settings such as mining and welding industries, through environmental overexposure due to contaminated drinking water, ephedrone abuse, end‐stage liver disease impairing biliary manganese excretion as well as overexposure through parenteral nutrition [51, 52, 53, 54]. Therefore, taking a thorough history is essential in patients with hypermanganesaemia to confirm the absence of excessive manganese exposure.

HMNDYT1 and 2 are progressive neurodegenerative disorders that, when left untreated, lead to significant neurodisability due to severe dystonia. Dysphagia and dysarthria are common. Complications of liver disease further contribute to morbidity and early mortality in HMNDYT1. The mainstay of treatment for both disorders to date remains intravenous chelation therapy with disodium calcium edetate (Na_2_CaEDTA) that facilitates mobilization of manganese from the liver and brain through urinary manganese excretion. Na_2_CaEDTA can reverse neurological symptoms to some degree and prevent disease progression, which can be monitored on MRI brain demonstrating a reduction in T1‐hyperintensity [16]. However, the unfavorable pharmacokinetics of Na_2_CaEDTA, including the absence of oral bioavailability and metal selectivity, make it a poor drug for clinical application, adding to the disease burden with recurrent hospitalization and long‐term venous access. The lack of availability of Na_2_CaEDTA and high associated healthcare costs deny many patients access to treatment. Alternative chelating agents have been trialed with some reported effect [50, 55, 56]. For HMNDYT1, iron supplementation can further reduce manganese stores due to competitive inhibition of both metals via shared transporters, with some patients having experienced a particularly favorable response [57]. Although available therapies are limited and burdensome, there is evidence that early treatment can prevent disease progression. We have developed consensus recommendations mainly based on expert opinion to facilitate early diagnosis and management for both HMNDYT1 and 2.

## 2 Materials and Methods

### 2.1 Development of a Consensus Expert Opinion

A consensus expert opinion was reached using the Delphi methodology [58, 59]. An international expert group was formed in 2021 consisting of pediatric and adult clinicians specialized in inborn errors of metabolism, neurology, hematology, genetics, and radiology to provide a representation across relevant specialities. Thirteen individuals from nine institutions across Europe, North America, Africa, and Asia were identified who had relevant clinical experience in the diagnosis or management of patients with HMNDYT. A list of statements was compiled based on available literature and the expertise of the group following an initial meeting in 2021, after which a survey was distributed among 111 clinicians and 13 societies representing the specialities metabolic medicine, neurology, hepatology, and hematology, with 31 responses received.

The survey consisted of 40 statements in total and each statement was evaluated using a 5‐point Likert scale as follows: completely agree, mostly agree, partially agree, mostly disagree, completely disagree. Consensus was defined as at least 66% agreement (completely agree or mostly agree) or disagreement (mostly disagree or completely disagree). Since HMNDYT1 and 2 are ultrarare diseases with only about 100 patients identified to date, the evidence for the recommendations is based on observational studies and expert opinions, representing low‐level quality evidence according to the GRADE system [60]. Similar to previous consensus recommendations, the strength of recommendations was defined as “strong” when there was at least 90% agreement (> 90% of respondents agree or completely agree) and “conditional” (< 90% of respondents agree or completely agree) [61].

Results from the initial survey were discussed in a meeting of the expert group in 2023. Four statements were revised, and a second survey was distributed among the 31 clinicians who returned the initial survey. This was followed by a further revision of three statements and a repeat survey in 2024. Consensus was reached for all 40 statements that have contributed to the final consensus expert opinion.

### 2.2 Systematic Literature Review

A systematic literature search of PubMed was performed for manuscripts published up to March 2024 (Table S1). Key search terms included SLC30A10 OR ZNT10 OR SLC39A14 OR ZIP14 AND manganese. A total of 140 results were reviewed and 27 clinically relevant articles were identified. Four additional articles from authors known to the expert group were added, resulting in a total of 31 papers reviewed.

## 3 Recommendations

### 3.1 Definition and Epidemiology

Statement 1 *HMNDYT1 and HMNDYT2 are rare diseases caused by biallelic pathogenic variants in the SLC30A10 and SLC39A14 genes, respectively. Biallelic pathogenic variants in these genes are associated with impaired excretion of manganese and result in the accumulation of manganese in the brain in both disorders, and additionally in the liver in HMNDYT1*.

*Level of evidence*: *observational studies—low quality*.

*Strength of recommendation*: *strong*.

*Expert opinion*: *Completely agree 90%, mostly agree 6.7%, partially agree 3.3%, mostly disagree 0%*, *completely disagree 4%*.

**FIGURE 3:**
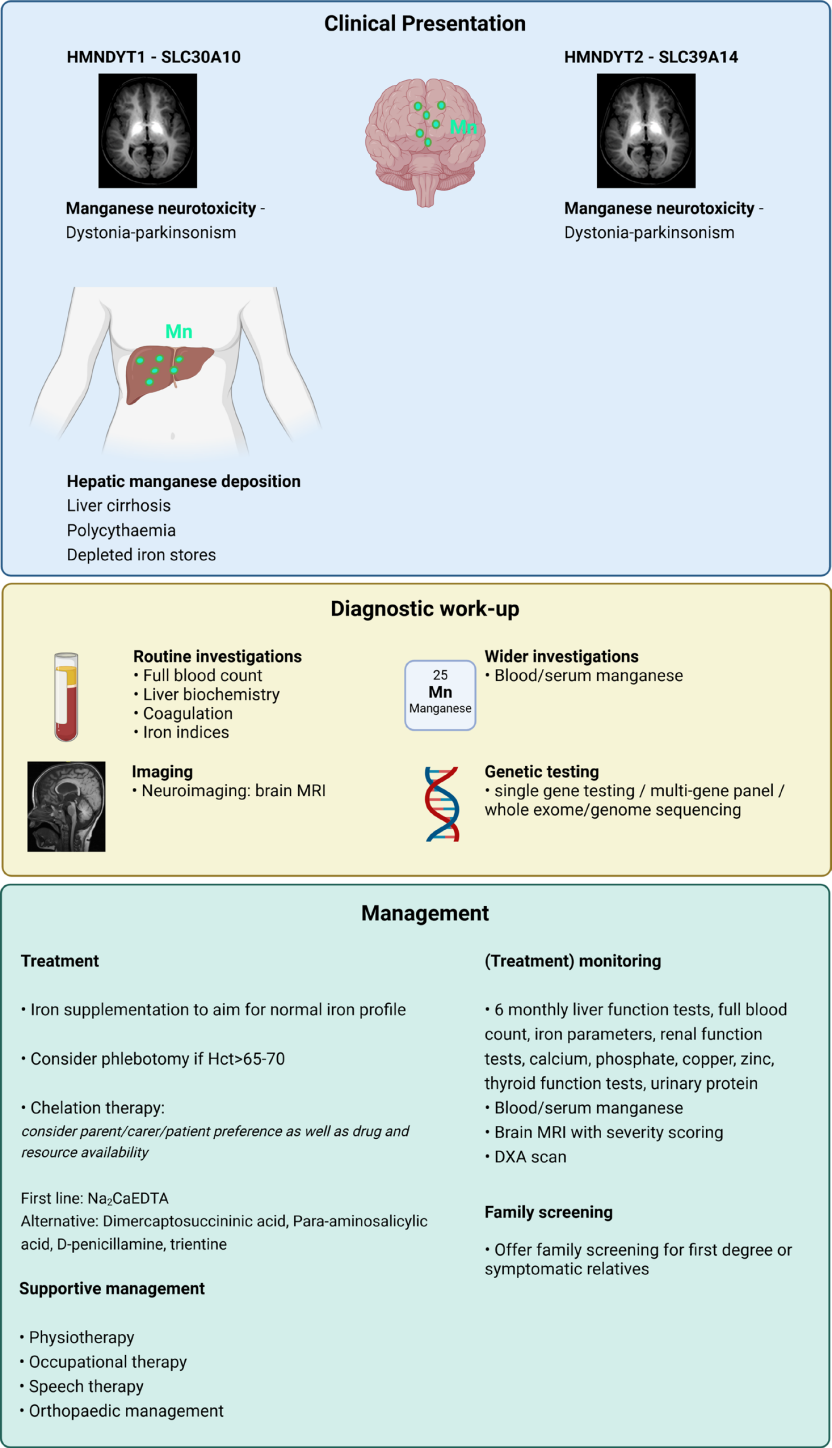
Investigations and management of HMNDYT1 and HMNDYT2. Created with BioRender.com.

High blood manganese and deposition of manganese in the brain, particularly the basal ganglia, are characteristic features of both HMNDYT1 and HMNDYT2 [7, 8, 14, 48, 49, 50, 56, 62, 63, 64]. In HMNDYT1, manganese also accumulates in the liver leading to cirrhosis and end‐stage liver failure (Figure 3) [7, 14, 48, 50, 56, 62, 64]. The clinical phenotypes of HMNDYT1 and HMNDYT2 are associated with pathogenic variants in *SLC30A10* and *SLC39A14*, respectively (Table S1) [7, 8, 14, 41, 48, 49, 50, 56, 62, 63, 64, 65].

Statement 2 *HMNDYT1 and HMNDYT2 are rare, panethnic diseases with an unknown incidence*.

*Level of evidence*: *observational studies—low quality*.

*Strength of recommendation*: *strong*.

*Expert opinion*: *Completely agree 90%, mostly agree 10%, partially agree 0%, mostly disagree 0%, completely disagree 0%*.

Our literature review demonstrates that HMNDYT1 and HMNDYT2 are prevalent across all continents and multiple ethnicities, with the highest numbers of affected individuals originating from India, United Arab Emirates, United States of America, and Brazil [41]. As of January 2025, a total of 98 individuals have been identified, 60 affected by HMNDYT1 and 33 by HMNDYT2. A further five patients with characteristic manganese neurotoxicity remain genetically undetermined (Table S1). One of the latter patients presents with polycythaemia and liver disease, three with liver involvement, and one with an isolated neurological phenotype.

### 3.2 Clinical Presentation

#### 3.2.1 HMNDYT1

Statement 3 *HMNDYT1 is a multisystem disorder involving the nervous system, liver*, *and blood*.

*Level of evidence*: *observational studies—low quality*.

*Strength of recommendation*: *strong*.

*Expert opinion*: *Completely agree 94%, mostly agree 6%, partially agree 0%, mostly disagree 0%, completely disagree 0%*.

The first patient with HMNDYT1 was described in 2000 while the disease‐causing gene was only identified in 2012 [7, 14, 43]. Affected individuals consistently present with dystonic movement disorders leading to a high‐stepping, “cock‐walk” gait with manganese deposition evident on brain MRI, accompanied by polycythaemia, and abnormal liver function tests [48]. The vast majority of patients diagnosed with HMNDYT1 present with dystonia, although one patient was asymptomatic [66]. All patients except for one presented with polycythaemia [67]. Liver involvement includes hepatomegaly and deranged liver function tests including hyperbilirubinaemia, elevated aspartate aminotransferase (AST), alanine aminotransferase (ALT) and alkaline phosphatase (ALP), and hypoalbuminaemia [48]. Liver ultrasound may identify hepatomegaly and increased parenchymal echogenicity. Liver biopsy can confirm a micronodular cirrhosis of the liver [64].

Statement 4 *HMNDYT1 is characterized by signs and symptoms of involvement of the central nervous system, in most cases by childhood‐onset, progressive dystonia‐parkinsonism. Intellectual ability is usually preserved*.

*Level of evidence*: *observational studies—low quality*.

*Strength of recommendation*: *strong*.

*Expert opinion*: *Completely agree 74%, mostly agree 23%, partially agree 3%, mostly disagree 0%, completely disagree 0%*.

The most frequent neurological phenotype consists of an early‐onset, rapidly progressive dystonic and parkinsonian movement disorder with the majority of patients manifesting before the age of 5 years [7, 14, 50, 56, 62]. For reasons that remain unknown, manganese accumulates in distinct, subcortical brain regions, particularly the globus pallidus, where it becomes neurotoxic and causes dystonia and parkinsonism [7, 8, 68]. While the integrity of dopaminergic neurons within the substantia nigra is preserved, evidenced by a normal Dopamine Transporter SPECT scan (DaTscan) in affected patients, rodent studies suggest that manganese interferes with the release of dopamine [16, 39, 40]. Multiple molecular mechanisms have been implicated in manganese neurotoxicity, including oxidative stress, mitochondrial dysfunction, calcium dyshomeostasis, and dysproteostasis [1, 2, 3, 69]. However, the hierarchy of these events remains to be elucidated.

Statement 5 *Rare presentations include spastic paraparesis and epilepsy*.

*Level of evidence*: *expert opinion—very low quality*.

*Strength of recommendation*: *strong*.

*Expert opinion*: *Completely agree 50%, mostly agree 43%, partially agree 3.5%, mostly disagree 3.5%, completely disagree 0%*.

One affected individual presented at the age of 14 years with spastic paraparesis without dystonic motor impairment [43]. Another individual presented with focal seizures without extrapyramidal motor or hepatic impairment. Given that seizures frequently occur in the pediatric population, further studies are required to prove a link between HMNDYT1 and epilepsy [70]. However, due to the rarity of the disorder, the full phenotypic spectrum of neurological manifestations has yet to be defined.

Statement 6 *Polycythaemia is a hallmark of HMNDYT1 and can be the presenting feature*.

*Level of evidence*: *observational studies—low quality*.

*Strength of recommendation*: *strong*.

*Expert opinion*: *Completely agree 71%, mostly agree 23%, partially agree 6%, mostly disagree 0%, completely disagree 0%*.

Manganese has been suggested to induce erythropoietin gene expression by mimicking the effect of hypoxia and is the likely mechanism behind the development of polycythaemia [7, 15, 55]. Of those patients who had full blood counts (FBC) measured at presentation, only one individual with genetically proven HMNDYT1 did not have polycythaemia [67]. This individual had other signs and symptoms of HMNDYT1 such as dystonia and elevated serum manganese at 186 mg/L (normal 5‐15 mg/L); his hemoglobin was 14.5 g/dL (normal 11.5–15.5 g/dL). There is increasing evidence that polycythaemia can precede the onset of neurological symptoms or hepatic disturbances, suggesting that HMNDYT1 should be considered in the differential diagnosis of polycthaemia [14, 41, 42]. For instance, one boy presented at the age of 12 months with polycythaemia and language delay [41].

Statement 7 *HMNDYT1 can present with liver disease*.

*Level of evidence*: *observational studies—low quality*.

*Strength of recommendation*: *strong*.

*Expert opinion*: *Completely agree 68%, mostly agree 26%, partially agree 6%, mostly disagree 0%, completely disagree 0%*.

Excess manganese deposited in the liver is hepatotoxic and leads to hepatic dysfunction which ranges from mild steatosis to fatal cirrhosis. Thirty‐nine of 65 individuals had liver involvement (Table S1). Two individuals with HMNDYT1 died due to complications of liver disease at the age of 18 and 46 years, respectively [14, 64]. Liver biopsy for several affected individuals showed micro‐ and macronodular cirrhosis with increased manganese tissue levels and positive Rhodanine staining [14, 64]. Hepatic manganese deposition can further be confirmed by MRI showing characteristic T1‐hyperintensity.

Statement 8 *Low iron status (raised total iron binding capacity, low ferritin) is a hematological characteristic of HMNDYT1*.

*Level of evidence*: *conditional*.

*Strength of recommendation*: *observational studies—low quality*.

*Expert opinion*: *Completely agree 58%, mostly agree 29%, partially agree 6.5%, mostly disagree 6.5%, completely disagree 0%*.

Regulation of manganese and iron homeostasis is closely interlinked since both metals are transported via shared transporters including the transferrin receptor, divalent metal transporter 1, and ferroportin [71]. This is corroborated by the finding that children with iron deficiency anemia have higher blood manganese levels compared to individuals with sufficient iron levels [72]. Of HMNDYT1 patients who had iron indices measured, all had low ferritin and raised total iron binding capacity (Table S1). Given that iron depletion is absent in HMNDYT2, it is plausible that the high manganese load in the liver is responsible for the changes in iron homeostasis. In keeping, Slc30a10 knockout mice show changes in expression levels of hepcidin, a hormone crucial for the regulation of intestinal iron absorption [15].

Statement 9 *HMNDYT1 can rarely present in adult life with prominent parkinsonism*.

*Level of evidence*: *strong*.

*Strength of recommendation*: *observational studies—low quality*.

*Expert opinion*: *Completely agree 66%, mostly agree 28%, partially agree 3%, mostly disagree 3%, completely disagree 0%*.

Quadri et al. reported two brothers born to first cousin parents who developed adult‐onset parkinsonism, hypermanganesemia, polycythaemia, and chronic liver disease [14]. The first individual was diagnosed at age 60 but had a 3‐year history of progressive gait disturbances and bradykinesia. He had presented at a much younger age with thrombocytopaenia and polycythaemia in his 30's. His younger brother presented at 59 years of age with a 12‐year history of gait disturbances, with polycythaemia diagnosed 2 years prior to this. Both brothers were found to carry homozygous pathogenic variants in the last exon of the *SLC30A10* gene affecting only the protein's C‐terminus, probably causing the milder phenotype [14].

#### 3.2.2 HMNDYT2

Statement 10 *HMNDYT2 is characterized by signs and symptoms of involvement of the central nervous system, most frequently by childhood‐onset, progressive dystonia–parkinsonism. Intellectual ability appears to be preserved*.

*Level of evidence*: *observational studies—low quality*.

*Strength of recommendation*: *strong*.

*Expert opinion*: *Completely agree 73%, mostly agree 20%, partially agree 7%, mostly disagree 0%, completely disagree 0%*.

Thirty‐two individuals with HMNDYT2 have been described in the literature to date. Onset of symptoms ranged between birth to 18 years, with a mean age of onset of 1 year and 9 months old with all but two patients presenting before or at the age of five (Table S1) [8, 49, 50, 63]. One individual who was evaluated at age 65 reported symptom onset at age 18, with handwriting changes along with impaired gait and balance [44].

These individuals generally presented with loss of developmental milestones, progressive dystonia, and bulbar dysfunction in infancy or early childhood. Disease progression was characterized by severe generalized dystonia, spasticity, limb contractures, scoliosis, and some parkinsonian features such as hypomimia, tremor, and bradykinesia [8]. There was cognitive sparing compared to motor disability [8, 49, 50, 63].

Of all individuals in the literature (33 in total), eight patients were reported to have cognitive impairment. Of those who had documented cognitive impairment, this included intellectual disability and learning disabilities, which may or may not affect individuals in the same family with the same pathogenic variants in *SLC39A14* [8, 49, 50, 63].

Statement 11 *Clinical liver disease and polycythaemia are not features of HMNDYT2*.

*Level of evidence*: *observational studies—low quality*.

*Strength of recommendation*: *strong*.

*Expert opinion*: *Completely agree 77%, mostly agree 17%, partially agree 6%, mostly disagree 0%, completely disagree 0%*.

Any literature to date has reported the absence of liver disease and polycythaemia in patients diagnosed with HMNDYT2 (Table S1). This is likely explained by the lack of manganese deposition in the liver [8].

### 3.3 Diagnostic Investigations

Statement 12 *All patients presenting with unexplained dystonia‐parkinsonism or other signs of central nervous dysfunction and suggestive brain MRI features of manganese deposition should have genetic testing for HMNDYT1 and HMNDYT2*.

*Level of evidence*: *observational studies—low quality*.

*Strength of recommendation*: *strong*.

*Expert opinion*: *Completely agree 90%, mostly agree 10%, partially agree 0%, mostly disagree 0%, completely disagree 0%*.

Both HMNDYT1 and HMNDYT2 are autosomal recessively inherited disorders [7, 14]. In order to confirm the diagnosis and allow family counseling, molecular analysis of the *SLC30A10* and *SLC39A14* genes is indicated using either single gene sequencing, multi‐gene panel analysis, or whole exome/genome sequencing.

To date, 32 pathogenic variants in *SLC30A10* and 22 pathogenic variants in *SLC39A14* have been reported in the literature (Table S1). Possible founder effects concern the c.1006C>T variant in *SLC30A10* identified in at least three families in Pakistan/India and the c.922C>T variant identified in a minimum of three families in Brazil (from personal communication with Prof Orlando Barsottini, Universidade Federal de São Paulo, Brazil) [7, 23, 50, 63, 73]. For *SLC39A14*, there appears to be one common pathogenic variant c.313G>T [8, 63, 74, 75]. Since blood manganese levels can fluctuate and, in rare cases, have been reported normal on some occasions, brain MRI is the most reliable indicator of the body's manganese load (See Statement 16) [14, 76]. Thus, if brain MRI shows manganese deposition, this should be followed up with genetic testing independent of the blood manganese level.

Statement 13 *In all patients with unexplained polycythaemia or liver disease*, *a diagnosis of HMNDYT1 should be considered and followed up with a blood Mn level and brain MRI*.

*Level of evidence*: *observational studies—low quality*.

*Strength of recommendation*: *strong*.

*Expert opinion*: *Completely agree 74%, mostly agree 19%, partially agree 7%, mostly disagree 0%, completely disagree 0%*.

Particularly, two recent reports have highlighted the importance of considering HMNDYT1 in the differential diagnosis of polycythaemia. Polycythaemia can indeed be the presenting symptom of HMNDYT1 preceding the manifestation of neurological symptoms or hepatic impairment [41, 42].

Statement 14 *Initial investigations should include measuring a blood manganese level, FBC (hemoglobin), iron parameters (TIBC, ferritin), liver function tests*, *and brain MRI*.

*Level of evidence*: *observational studies—low quality*.

*Strength of recommendation*: *strong*.

*Expert opinion*: *Completely agree 90%, mostly agree 10%, partially agree 0%, mostly disagree 0%, completely disagree 0%*.

A raised blood manganese level is a characteristic of both HMNDYT1 and HMNDYT2, which is accompanied by manganese deposition in the brain, as demonstrated on MRI brain shared by all patients reported in the literature (Table S1) [48, 49]. Polycythaemia, depleted iron stores, and abnormal liver function tests allow differentiation between HMNDYT1 and HMNDYT2 since only HMNDYT1 shows non‐neurological features due to hepatic manganese deposition [7, 14]. Therefore, HMNDYT1 and HMNDYT2 have clear biochemical markers that, together with MRI brain, enable early diagnosis of affected individuals.

Statement 15 *Manganese accumulation in the brain causes characteristic brain MRI abnormalities. These include hyperintensity of the globus pallidus and striatum, dentate nucleus, cerebral white matter*, *and dorsal pons with characteristic sparing of the ventral pons on T1 weighted imaging with/without hypointensity of the corresponding regions on T2 weighted imaging*.

*Level of evidence*: *observational studies—low quality*.

*Strength of recommendation*: *strong*.

*Expert opinion*: *Completely agree 90%, mostly agree 10%, partially agree 0%, mostly disagree 0%, completely disagree 0%*.

Manganese is a paramagnetic metal and acts as a contrast agent for MRI by shortening the T1‐relaxation time [77]. Manganese deposition in the brain can therefore be readily seen on T1‐weighted MR imaging (Figure 2). Although MRI changes are significant, manganese‐related MRI brain appearances have been missed by clinicians in the past (from clinical experience by Dr. Karin Tuschl, Great Ormond Street Hospital for Children, London, UK); therefore, it is crucial to raise the awareness across clinical specialties to ensure early diagnosis of patients with HMNDYT1/2. Since MRI signal intensity of the basal ganglia changes with aging, involvement of a paediatric neuroradiologist is advised to recognize subtle changes of manganese deposition that may be interpreted as normal in the older population [78].

Statement 16 *Brain MRI is the most reliable diagnostic tool (for severity scoring see statement 37). Blood manganese in both HMNDYT1 and HMNDYT2, as well as hemoglobin and liver function tests in HMNDYT1 can be normal, particularly in adulthood*.

*Level of evidence*: *expert opinion—very low quality*.

*Strength of recommendation*: *conditional*.

*Expert opinion*: *Completely agree 60.5%, mostly agree 25%, partially agree 11%, mostly disagree 3.5%, completely disagree 0%*.

Several patients with HMNDYT1 have been reported to have normal liver function tests, which is likely dependent on the hepatic involvement due to age and disease progression (Table S1). Occasionally, in some patients with HMNDYT1, manganese levels reduced significantly over time, with two patients showing only a minimally raised blood manganese level of 41.7 nmol/L (reference range < 32.8) or complete normalization during adult life [14, 76]. Since iron supplementation, for instance, can dramatically reduce manganese levels, dietary changes and supplements will have a significant impact on manganese levels as well as red blood cell count [57, 64]. Therefore, a normal or mildly raised blood manganese or hemoglobin level may not completely rule out a diagnosis of HMNDYT1 or HMNDYT2. Brain manganese deposition, on the other hand, is thought to take several months to reduce, even on chelation treatment [16]. Therefore, brain manganese detected by MRI is likely to more accurately reflect the body's manganese store, suggesting it to be a more reliable marker to assess systemic manganese overload.

Statement 17 *Genetic testing is the only reliable method for carrier screening of family members and prenatal diagnosis*.

*Level of evidence*: *observational studies—low quality*.

*Strength of recommendation*: *strong*.

*Expert opinion*: *Completely agree 87%, mostly agree 13%, partially agree 0%, mostly disagree 0%, completely disagree 0%*.

Some heterozygous carrier individuals have shown mildly elevated blood manganese levels, but others have shown normal levels [7, 8, 43]. Therefore, genetic testing remains the only reliable investigation to prove carrier status.

Statement 18 *Genetic counseling should be offered to families of affected individuals*.

*Level of evidence*: *observational studies—low quality*.

*Strength of recommendation*: *strong*.

*Expert opinion*: *Completely agree 94%, mostly agree 6%, partially agree 0%, mostly disagree 0%, completely disagree 0%*.

### 3.4 Diagnostic Criteria

Statement 19 *Probable diagnostic criteria are signs and symptoms of involvement of the central nervous system (*e.g., *dystonia‐parkinsonism) associated with raised blood manganese and brain MRI abnormalities which include hyperintensity of the globus pallidus and striatum, dentate nucleus, cerebral white matter*, *and dorsal pons with characteristic sparing of the ventral pons on T1 weighted imaging with/without hypointensity of the corresponding regions on T2 weighted imaging. In addition, probable diagnostic criteria for HMNDYT1 include polycythaemia, liver disease*, *and depleted iron stores*.

*Level of evidence*: *observational studies—low quality*.

*Strength of recommendation*: *strong*.

*Expert opinion*: *Completely agree 87%, mostly agree 10%, partially agree 3%, mostly disagree 0%, completely disagree 0%*.

The characteristic constellation of features of a neurological phenotype associated with the pathognomonic MRI brain appearances of manganese deposition should prompt any clinician to consider a diagnosis of HMNDYT1 or HMNDYT2 since almost all patients to date have followed this clinical presentation (Table S1). For HMNDYT1, hepatic manganese accumulation results in the additional features of polycythaemia, cirrhosis, and iron depletion (Figure 3) [7, 14].

Statement 20 *Definite diagnostic criteria are the presence of biallelic pathogenic variants in the SLC30A10 and SLC39A14 genes consistent with the diagnosis of HMNDYT1 and HMNDYT2, respectively*.

*Level of evidence*: *observational studies—low quality*.

*Strength of recommendation*: *strong*.

*Expert opinion*: *Completely agree 84%, mostly agree 13%, partially agree 3%, mostly disagree 0%, completely disagree 0%*.

### 3.5 Principles of Treatment

Statement 21 *All patients with HMNDYT1 should be treated with iron supplementation aiming for a normal iron profile. This should exclude individuals with a transferrin saturation of > 45%. (Optimizing iron stores will reduce Mn absorption/accumulation due to interdependence of Mn and iron homeostasis)*.

*Level of evidence*: *observational studies—low quality*.

*Strength of recommendation*: *strong*.

*Expert opinion*: *Completely agree 63%, mostly agree 30%, partially agree 7%, mostly disagree 0%, completely disagree 0%*.

Several case reports have demonstrated the benefit of oral iron supplementation in reducing blood manganese levels in individuals affected with HMNDYT1, with some patients experiencing dramatic improvement of motor symptoms [57, 64, 73]. Optimizing iron stores is expected to reduce manganese absorption and accumulation due to the interdependence of manganese and iron homeostasis [71]. This does not apply to HMNDYT2 patients; neither do they show the typical features of iron depletion, suggesting that the hepatic manganese deposition observed in HMNDYT1 is the driver of iron dyshomeostasis. Monitoring iron indices is advised in order to prevent possible iron toxicity.

Statement 22 *Consider phlebotomy if Hct > 65–70*.

*Level of evidence*: *expert opinion—very low*.

*Strength of recommendation*: *strong*.

*Expert opinion*: *Completely agree 73%, mostly agree 20%, partially agree 7%, mostly disagree 0%, completely disagree 0%*.

To date, no thromboembolic events have been reported in HMNDYT1 patients who can experience pronounced polycythaemia with hemoglobin values exceeding 20 g/dL [7, 50]. However, several patients have undergone repeat phlebotomy to reduce the risk of thromboembolism (Table S1). While the mainstay of treatment for polycythaemia vera, the benefit of phlebotomy in HMNDYT1 remains unclear, similar to the application of phlebotomy in secondary polycythaemia [79].

Statement 23 *Treatment should be decided in consideration of parent/carer/patient preference as well as drug and resource availability*.

*Level of evidence*: *expert opinion—very low*.

*Strength of recommendation*: *strong*.

*Expert opinion*: *Completely agree 90%, mostly agree 7%, partially agree 0%, mostly disagree 3%, completely disagree 0%*.

Chelation therapy with Na_2_CaEDTA follows a burdensome treatment regimen due to intravenous administration and is associated with high healthcare costs. Furthermore, the availability of Na_2_CaEDTA is limited in some countries [55, 56]. Therefore, discussions should be led with affected individuals and their families to decide whether Na_2_CaEDTA therapy is in the best interest of the patient.

Statement 24 *All patients with symptomatic HMNDYT1 and HMNDYT2 should be offered chelation therapy if locally available*.

*Level of evidence*: *observational studies—low quality*.

*Strength of recommendation*: *strong*.

*Expert opinion*: *Completely agree 84%, mostly agree 13%, partially agree 0%*, *mostly disagree 3%, completely disagree 0%*.

Chelation therapy is the mainstay of treatment for metal toxicity. Binding of a metal to the chelating agent results in the formation of chelates which are complex ring structures that are subsequently excreted in the urine, thereby reducing the body's metal load [80]. Chelation therapy has been shown to effectively reduce brain manganese deposition in both HMNDYT1 and HMNDYT2, which is accompanied by improvement in neurological symptoms and halt of liver disease progression in HMNDYT1 [7, 8, 14, 50, 64]. Improvement of neurological symptoms demonstrates that some aspects of manganese neurotoxicity are reversible despite post mortem studies showing neurodegenerative changes within the basal ganglia, particularly the globus pallidus [76]. This is in keeping with observations in animal models of HMNDYT1 and HMNDYT2, suggesting functional deficiencies upon manganese neurotoxicity such as impaired neurotransmitter release that seem to occur prior to the development of cell death [39, 40, 81].

Statement 25 *If available, patients with symptomatic HMNDYT1 and HMNDYT2 should commence on intravenous chelation therapy with Na*_*2*_ *CaEDTA*.

*Level of evidence*: *observational studies—low quality*.

*Strength of recommendation*: *strong*.

*Expert opinion*: *Completely agree 87%, mostly agree 10%, partially agree 0%, mostly disagree 3%, completely disagree 0%*.

The majority of patients treated with chelation therapy to date were commenced on Na_2_CaEDTA. There is significant evidence that both HMNDYT1 and HMNDYT2 patients have benefitted from Na_2_CaEDTA therapy with significant improvement of dystonia and motor symptoms (Table S1) [7, 8, 14, 50, 64, 74]. Reduction of manganese brain deposition is evident on MRI brain [16].

Statement 26 *The treatment regimen of Na*_*2*_ *CaEDTA should be adjusted to clinical response and patient preference. The two common regimens used are monthly five‐day courses of twice‐daily infusions or a weekly single infusion*.

*Level of evidence*: *expert opinion—very low*.

*Strength of recommendation*: *strong*.

*Expert opinion*: *Completely agree 74%, mostly agree 19.5%, partially agree 6.5%, mostly disagree 0%, completely disagree 0%*.

The first attempt of chelation therapy with Na_2_CaEDTA was in a patient with HMNDYT1. A single, five‐day course of parenteral Na_2_CaEDTA (following the treatment guidelines for lead exposure in children by the American Academy of Pediatrics Committee on Drugs [82]) led to a significant increase of urinary manganese excretion. Subsequently, the patient received monthly, five‐day courses of Na_2_CaEDTA which led to the improvement of dystonia reflected in the normalization of gait and improved fine motor movements [7, 16, 64]. Over 9 years of treatment with Na_2_CaEDTA, T1 hyperintensity on MRI brain had much improved confirming the successful removal of manganese [16]. In order to lessen the burden of monthly, five‐day hospital admissions, some patients were changed to once‐weekly infusions of Na_2_CaEDTA with a similar effect (from personal communication with Dr. Vala Palmadottir, Landspitali University Hospital, Iceland).

Statement 27 *Per infusion, the recommended dose of Na*_*2*_ *CaEDTA is 20 mg/kg/dose made up in 250 mL of 0.9% sodium chloride and given intravenously over 1 h*.

*Level of evidence*: *observational studies—low quality*.

*Strength of recommendation*: *strong*.

*Expert opinion*: *Completely agree 82%, mostly agree 14%, partially agree 4%, mostly disagree 0%, completely disagree 0%*.

This dosing is based on the treatment guidelines for lead exposure in children by the American Academy of Pediatrics Committee on Drugs [82].

Statement 28 *There is limited evidence for treatment with alternative chelation agents (to Na*_*2*_ *CaEDTA) including Penicillamine, 2,3 Dimercaptosuccinic acid, Para‐aminosalicylic acid*, *and Trientine. However, if Na*_*2*_ *CaEDTA is unavailable or not tolerated*, *these chelators can be considered*.

*Level of evidence*: *expert opinion—very low quality*.

*Strength of recommendation*: *conditional*.

*Expert opinion*: Completely agree 40.7%, mostly agree 40.7%, partially agree 18.5%, mostly disagree 0%, completely disagree 0%.

With the lack of relevant clinical trial data available, there is limited evidence that the above chelation agents are efficient in chelating manganese ions and reducing brain manganese deposition. While some case reports suggest that D‐Penicillamine can lead to improvement of neurological symptoms to some extent, urinary excretion of manganese in response to D‐Penicillamine is minimal [55, 64, 83]. 2,3 Dimercaptosuccinic acid (DMSA) has been reported to improve blood manganese levels and motor symptoms in nine patients with HMNDYT1, particularly in those with milder neurological phenotypes [56]. However, of note, these patients were also given iron supplementation; hence, no conclusion can be made regarding the effectiveness of DMSA. Para‐aminosalicylic acid (PAS) has been shown effective in treating environmental manganese intoxication in some cases [84]. There is limited experience of the use of PAS in inherited manganese transporter defects. A single attempt with a short course of PAS in treating a patient with HMNDYT1 did not lead to apparent clinical improvement (personal communication Dr. Maude Landry) [85, 86]. Studies in both neuronal culture and rodent models suggest that PAS may have a dual action and act both as a chelator of manganese as well as an antioxidant [87, 88, 89]. These characteristics, together with its ability to cross the blood–brain barrier and oral bioavailability, make PAS a promising compound for further studies [87]. There is a single case report of a patient with HMNDYT1 presenting predominantly with polycythaemia who was treated with Trientine with subsequent normalization of hemoglobin and reduction in serum manganese, accompanied by improved MRI brain appearances suggesting a reduction in Mn deposition [42].

Statement 29 *A therapeutic Mn restricted diet should not be pursued in orally feeding patients. It may be applied in gastrostomy‐fed patients*.

*Level of evidence*: *expert opinion—very low quality*.

*Strength of recommendation*: *conditional*.

*Expert opinion*: *Completely agree 59%, mostly agree 24%, partially agree 14%, mostly disagree 3%, completely disagree 0%*.

Dietary restriction of manganese has been attempted in a patient with HMNDYT2 who received manganese‐free gastrostomy feeds. However, no effect on blood manganese levels was observed. This questions whether dietary manganese restriction is beneficial. Manganese is ubiquitously found in the diet; however, some foods are particularly high in manganese, including nuts, chocolate, shellfish, fruits, cereals, and tea [90]. Therefore, an oral diet restricted in manganese is difficult to establish.

### 3.6 Disease and Treatment Monitoring

Statement 30 *Chelation therapy for presymptomatic individuals with biallelic variants may be considered*.

*Level of evidence*: *expert opinion—very low quality*.

*Strength of recommendation*: *conditional*.

Expert opinion: Completely agree 40.7%, mostly agree 40.7%, partially agree 18.5%, mostly disagree 0%, completely disagree 0%.

Similar to Wilson's disease, it seems logical that chelation therapy in presymptomatic individuals with HMNDYT1 and HMNDYT2 would remove neurotoxic manganese and prevent disease onset [91]. However, due to the invasive nature and possible adverse effects of Na_2_CaEDTA, it remains debatable when treatment should be initiated. This is further complicated by the fact that milder phenotypes exist with disease manifestation during adolescence or even adulthood [14].

Statement 31 *Assessment for physiotherapy, occupational therapy, speech therapy, and orthopedic management to prevent contractures and maintain ambulation should be pursued as required*.

*Level of evidence*: *observational studies—low quality*.

*Strength of recommendation*: *strong*.

*Expert opinion*: *Completely agree 100%, mostly agree 0%, partially agree 0%, mostly disagree 0%, completely disagree 0%*.

As with any childhood disorder causing neurodisability, appropriate supportive care is essential.

Statement 32 *Disease monitoring should include at least monthly assessment of liver function tests, FBC (Hb) and iron parameters during the initial presentation. Once the disease course has stabilized*, *the interval can be extended to 6 monthly*.

*Level of evidence*: *observational studies—low quality*.

*Strength of recommendation*: *strong*.

*Expert opinion*: *Completely agree 77%, mostly agree 20%, partially agree 3%, mostly disagree 0%, completely disagree 0%*.

This is particularly relevant to HMNDYT1 that presents with liver disease, polycythaemia, and depleted iron stores and will allow timely intervention with e.g., phlebotomy, iron supplementation, and hepatology assessment.

Statement 33 *Severity of movement disorder should be monitored using a clinical rating scale with video documentation at baseline and follow up*.

*Level of evidence*: *observational studies—low quality*.

*Strength of recommendation*: *strong*.

*Expert opinion*: *Completely agree 90%, mostly agree 10%, partially agree 0%, mostly disagree 0%, completely disagree 0%*.

Multiple clinical assessment tools have been developed to objectively measure the severity of dyskinetic movement disorders [92]. Regular assessment will allow to determine the progression of neurological symptoms and response to therapeutic intervention.

Statement 34 *Blood/serum Mn levels can be monitored if metal‐free blood sampling tubes and appropriate diagnostic laboratories are available*.

*Level of evidence*: *observational studies—low quality*.

*Strength of recommendation*: *conditional*.

*Expert opinion*: *Completely agree 61%, mostly agree 28%, partially agree 11%, mostly disagree 0%, completely disagree 0%*.

In order to avoid contamination from the environmental presence of metals, trace element analysis is ideally carried out in metal‐free blood sampling tubes and laboratories familiar with metallomics workflows. This may be circumvented by the analysis of an empty blood bottle from the same batch to detect any background contamination. However, since blood manganese levels do not necessarily reflect tissue levels and clinical management does not change upon fluctuations in blood manganese, pursuing regular blood manganese levels may not be required in low‐income settings without availability of appropriate metallomics laboratories [64].

Statement 35 *Brain MRI most reliably assesses the body's Mn load and should be used to assess* the *efficacy of treatment. Frequency of imaging depends on disease course and should be decided on an individual basis and resource availability*.

*Level of evidence*: *observational studies—low quality*.

*Strength of recommendation*: *strong*.

*Expert opinion*: *Completely agree 59%, mostly agree 34%, partially agree 7%, mostly disagree 0%, completely disagree 0%*.

Due to its paramagnetic properties, brain manganese deposition can readily be detected on T1‐weighted MRI [93]. Mobilization of manganese from brain tissue can therefore be reliably monitored, showing a significant decrease in T1 hyperintensity after several months of chelation therapy [16, 47].

Statement 36 *Severity scoring of brain MRI changes can be performed using the following qualitative scoring*:

*Evaluate each structure with a score from 0 to 2 depending on* the *severity of T1 increase*.

Maximum score 28.

*0 = normal, 1 = mildly increased, 2 = greatly increased T1 signal*.

*Structures to evaluate*: *globus pallidus–interna and externa, putamen, caudate, internal capsule, external capsule, thalamus, deep white matter, subcortical white matter, midbrain, dorsal pons, medulla, superior cerebellar peduncles, middle cerebellar peduncles*.

Level of evidence: expert opinion—very low quality.

*Strength of recommendation*: *strong*.

*Expert opinion*: *Completely agree 58%, mostly agree 42%, partially agree 0%, mostly disagree 0%, completely disagree 0%*.

After neuroradiology review of all brain MR imaging from HMNDYT1 and HMNDYT2 patients available within our clinician expert group and in the literature, we propose the above scoring scheme to quantify the extent of manganese deposition in the brain that will allow the assessment of disease progression and efficacy of treatment.

Statement 37 *Treatment monitoring of chelation therapy should include FBC, renal function (including testing for proteinuria) and electrolytes, Ca, Mg, Phosphate, Cu*, *and Zn (depending on resource availability) before and after chelation cycles*.

*Level of evidence*: *observational studies—low quality*.

*Strength of recommendation*: *strong*.

*Expert opinion*: *Completely agree 80%, mostly agree 17%, partially agree 3%, mostly disagree 0%, completely disagree 0%*.

Chelating agents currently used in the clinic, mainly CaNa_2_EDTA, are not manganese specific. Therefore, there is a risk of loss of other essential minerals including zinc, iron, calcium, magnesium, and phosphate [94]. CaNa_2_EDTA can also exert nephrotoxicity and cause leuko‐ and thrombocytopenia. Therefore, monitoring of urinary protein as well as FBC is recommended [48, 49].

Statement 38 *Treatment of iron supplementation should be monitored with iron parameters (TIBC and ferritin) every 3 months to avoid iron overload*.

*Level of evidence*: *expert opinion—very low quality*.

*Strength of recommendation*: *strong*.

*Expert opinion*: *Completely agree 77%, mostly agree 20%, partially agree 3%, mostly disagree 0%, completely disagree 0%*.

Due to the benefit of iron supplementation on the body's manganese load due to the interdependence of iron and manganese transport, iron supplementation is recommended with the view to keep iron indices within the high normal range. However, since limited evidence is available as to whether continuous iron supplementation may lead to iron toxicity, we suggest regular monitoring of iron indices.

Statement 39 *A dual‐energy X‐ray absorptiometry (DXA) scan to assess bone health should be performed in patients receiving regular chelation therapy since fractures have been reported as complications of chelation therapy*.

*Level of evidence*: *expert opinion—very low evidence*.

*Strength of recommendation*: *conditional*.

Expert opinion: Completely agree 59.3%, mostly agree 25.9%, partially agree 14.8%, mostly disagree 0%, completely disagree 0%.

We are aware of a case of spinal fracture with reduced bone density in a patient with HMNDYT1 who has been on long‐term chelation therapy with CaNa_2_EDTA (personal communication). Since EDTA can chelate calcium and lead to hypocalcaemia, we recommend assessing bone health in patients undergoing continuous chelation therapy [94].

Statement 40 *Thyroid function tests should be monitored annually as hypothyroidism has been reported in one patient with HMNDYT1 and rodent models of HMNDYT1*.

*Level of evidence*: *expert opinion—very low evidence*.

*Strength of recommendation*: *strong*.

*Expert opinion*: *Completely agree 60%, mostly agree 30%, partially agree 10%, mostly disagree 0%, completely disagree 0%*.

Slc30a10 knockout in mice has been shown to lead to profound hypothyroidism likely due to impaired thyroxine production with associated high thyroid stimulating hormone (TSH) [95]. While primary hypothyroidism has only been described in one patient with HMNDYT1, it seems indicated to monitor thyroid function tests in patients presenting with systemic manganese overload.

## 4 Conclusions

We provide consensus expert recommendations for the diagnosis, treatment, and monitoring of patients affected by the inherited manganese transporter defects HMNDYT1 and HMNDYT2. Since these are only recently identified, ultrarare diseases, the awareness of HMNDYT1 and HMNDYT2 among clinicians, particularly their characteristic MRI brain appearances, is limited. With the rapid implementation of next generation sequencing in all parts of the world, it is expected that the number of patients presenting with HMNDYT1 and HMNDYT2 will expand considerably. Given that chelation therapy and iron supplementation can effectively halt disease progression in both disorders, we suggest a standardized protocol for diagnosis and clinical management. The development of novel treatments such as genetic therapies and manganese specific chelators is likely to improve clinical outcome for HMNDYT1 and HMNDYT2 in the future.

The provided consensus recommendations are heavily based on expert opinions. Therefore, continuous evaluation of these recommendations, particularly in the absence of clinical trials for ultrarare diseases, will be essential to optimize patient care.

## Author Contributions

Systematic literature review: Karin Tuschl, Sherry Fang. Development of statements: Karin Tuschl, Sangeetha Yoganathan, Maha S. Zaki, Elin A. Helgadottir, Vala K. Palmadottir, Maude Landry, Sidney M. Gospe, Kshitij Mankad, Peter T. Clayton, Vincenzo Bonifati, Divyani Garg, Suvasini Sharma. Manuscript draft: Sherry Fang, Karin Tuschl. Review of final manuscript: Sherry Fang, Sangeetha Yoganathan, Maha S. Zaki, Elin A. Helgadottir, Vala K. Palmadottir, Maude Landry, Sidney M. Gospe, Kshitij Mankad, Peter T. Clayton, Vincenzo Bonifati, Divyani Garg, Suvasini Sharma, Karin Tuschl.

## Consent

The authors have nothing to report.

## Conflicts of Interest

The authors declare no conflicts of interest.

## Supporting information


**Table S1.** List of patients with a diagnosis of HMNDYT1 or 2 published in the literature to date. Clinical details are provided including genetic variants, clinical symptoms, biochemical and imaging features as well as trialed treatments.
